# Clinical study investigating the role of lymphadenectomy, surgical castration and adjuvant hormonal treatment in endometrial stromal sarcoma

**DOI:** 10.1038/sj.bjc.6603986

**Published:** 2007-09-25

**Authors:** F Amant, A De Knijf, B Van Calster, K Leunen, P Neven, P Berteloot, I Vergote, S Van Huffel, P Moerman

**Affiliations:** 1 Division of Gynecological Oncology, Leuven Cancer Institute (LKI), Department of Obstetrics and Gynecology, UZ Gasthuisberg, Katholieke Universiteit Leuven, Leuven, Belgium; 2 Department of Electrical Engineering, ESAT, Katholieke Universiteit Leuven, Leuven, Belgium; 3 Department of Pathology, UZ Gasthuisberg, Katholieke Universiteit Leuven, Leuven, Belgium

**Keywords:** endometrial, stromal, sarcoma, adenosarcoma, hormonal, lymphadenectomy

## Abstract

The objective of this study is to assess the therapeutic importance of surgical castration, adjuvant hormonal treatment and lymphadenectomy in endometrial stromal sarcoma (ESS). A retrospective and multicentric search was performed. Clinicopathologic data were retrieved from cases that were confirmed to be ESS after central pathology review. The protocol was approved by the Ethical Committee. ESS was confirmed histopathologically in 34 women, but follow-up data were available in only 31 women. Surgical treatment (*n*=31) included hysterectomy with or without bilateral salpingo-oophorectomy (BSO) in 23 out of 31 (74%) and 8 out of 31 (26%) cases, respectively. Debulking surgery was performed in 6 out of 31 cases (19%). Stage distribution was as follows: 22 stage I, 4 stage III and 5 stage IV. Women with stage I disease recurred in 4 out of 22 (18%) cases. Among stage I women undergoing hormonal treatment with or without BSO, 3 out of 15 (20%) and 1 out of 7 (14%) relapsed, respectively. Among stages III–IV women receiving adjuvant hormonal treatment or not, 1 out of 5 (20%) and 3 out of 4 (75%) relapsed, respectively (differences=55.0%, 95% CI=−6.8–81.2%). Kaplan–Meier curves show comparable recurrence rates for stage I disease without adjuvant hormonal treatment when compared to stages III–IV disease treated with surgery and adjuvant hormonal treatment. Furthermore, women taking hormones at diagnosis have a better outcome when compared to women not taking hormonal treatment. Three out of 31 (9%) patients had a systematic lymphadenectomy whereas 3 out of 31 (9%) had a lymph node sampling. In one case, obvious nodal disease was encountered at presentation. Isolated retroperitoneal recurrence occurred in 1 out of 31 (3%) of all cases and in 1 out of 8 (13%) recurrences. This single woman later also developed lung and abdominal metastases. Leaving lymph nodes *in situ* does not appear to alter the clinical outcome of ESS. Although numbers are low, the retrospective data suggest that the need for surgical castration (BSO) in premenopausal women with early-stage disease should be discussed with the patient on an individual basis. The data support the current practice in some centres to administer adjuvant hormonal treatment.

Endometrial stromal sarcomas (ESSs) are composed of cells that resemble proliferative phase or hyperplastic endometrial stromal cells (Hart and Yoonnessi, 1977; Evans, 1982). Currently, the designation ESS is restricted to malignancies that were formally referred to as low-grade stromal sarcomas (Evans, 1982; Oliva *et al*, 2000). Endometrial sarcomas without recognisable evidence of a definite endometrial stromal phenotype, designated as poorly differentiated endometrial sarcomas, are almost invariably high grade (Evans, 1982; Oliva *et al*, 2000), and termed poorly differentiated or undifferentiated uterine sarcomas. The classification of a uterine sarcoma as ‘high-grade endometrial stromal sarcoma’ should therefore be abandoned (Amant *et al*, 2004a). Here, we focus on ESS, excluding endometrial stromal nodules and undifferentiated or poorly differentiated uterine sarcomas.

The rarity of ESS, the historical mixture in studies with poorly differentiated or undifferentiated uterine sarcomas and the recent classification of ESS as a separate entity result in limited data on ‘keypoint’ issues, including standard treatment protocols.

Lymphatic invasion is well-established and pathognomonic for ESS, formerly designated as endolymphatic stromal myosis. Despite this, lymph node involvement is not considered a clinical problem and pelvic lymphadenectomy is not added to hysterectomy as the cornerstone of the treatment. However, recent data suggested the incidence of lymph node metastases to be higher than suspected (Riopel *et al*, 2005), suggesting the need for a more extensive lymph node sampling (Riopel *et al*, 2005).

Almost all ESSs show oestrogen and progesterone receptors (Reich *et al*, 2000), rendering these tumours hormone sensitive. Endometrial stromal sarcoma development and/or recurrences can be induced by oestrogen-replacement therapy and tamoxifen (Beer *et al*, 1995; Chu *et al*, 2003), whereas progestins (Gloor *et al*, 1982), gonadotropin-releasing hormone analogues (Burke and Hickey, 2004) and aromatase inhibitors inhibit tumour growth (Maluf *et al*, 2001; Leunen *et al*, 2004). Recurrence rates in early-stage ESS are high (Chang *et al*, 1990), and the impact of surgical castration and adjuvant hormonal treatment needs to be investigated further.

Given the paucity of data on standard treatment modalities, we aimed to explore the value of lymphadenectomy and surgical/medicinal modification of hormonal levels.

## MATERIALS AND METHODS

After approval of the study protocol by the Local Ethical Committee, we retrospectively searched for ESS cases from 1986 to 2005, in a multicentric study with the following hospitals: UZ Gasthuisberg Leuven, Institut Bordet Brussels, St Maria ziekenhuis Halle, Stedelijk ziekenhuis Roeselare, AZ Groeninge Kortrijk, AZ St Jan Genk, St Jozef ziekenhuis Malle, CHU Liège, M Middelares ziekenhuis St Niklaas. Representative slides were submitted for central pathology review (PM).

The following information was retrieved through a search of hospital charts: age, menopausal status, hormonal intake at diagnosis, symptoms, delay in diagnosis, surgical treatment, stage, adjuvant hormonal treatment, and recurrence and survival follow-up information. Staging was retrospectively based on the surgical and pathology reports and according to the endometrial cancer FIGO staging (Amant *et al*, 2005). Postoperative progestin dosages of minimum 200 mg day^−1^ were accepted as therapeutic, lower dosages were considered as no adjuvant hormonal treatment. If the patient recurred, the time from diagnosis until recurrence was recorded. If the patient did not recur to date, the follow-up time (from diagnosis until the end of follow-up for the patient) was recorded. We are certain that the patient did not recur during this period. Survival follow-up information was collected analogously, with death rather than recurrence being the event of interest.

### Statistical analysis

Due to the small sample size and general problems with significance testing (Sterne and Davey Smith, 2001), we did not use tests for statistical significance. Also, even though our study has more data than other studies on this topic, the sample is too small to draw firm conclusions. We can only investigate what our data suggest and whether our results corroborate previous findings or hypotheses.

When comparing proportions, we reported the difference in proportions together with a 95% confidence interval (CI) on this difference using method 10 from Newcombe (1998). Recurrence data were used to generate Kaplan–Meier curves (Kaplan and Meier, 1958) to visualise the estimated probability of recurrence at different time points relative to the moment of surgery. Again, we did not use any test (such as the Wilcoxon test) to test for statistically significant differences between Kaplan–Meier curves.

All analyses were performed using SAS v9.1 (SAS Institute, Cary, NC, USA).

## RESULTS

We collected 34 women with ESS. One patient was excluded because of concomitant breast cancer. Since one patient was lost to follow-up and one patient also developed breast cancer during follow-up, data on recurrence and survival were based on 31 women who had a hysterectomy and adequate follow-up data.

The median age at diagnosis was 44 years (range: 18–60 years). Women were premenopausal in 25 out of 31 (81%) of cases. The distribution of symptoms was as follows (more than one symptom per patient possible): menstrual disorder (21, 68%), postmenopausal bleeding (4, 13%), dyspareunia (4, 13%), abdominal mass or pain (3, 10%), prolapsing polyp (1, 3%) and excessive postpartum bleeding (1, 3%). Two patients (6%) were asymptomatic and an enlarged uterus was found during yearly gynaecological preventive control. In one patient, diagnosis was based on an accidental finding of a lung metastasis. Seven out of seven women (100%) who received hormonal treatment at diagnosis had stage I disease, while only 15 out of 24 (63%) women not receiving hormones had stage I disease (difference=37.5%, 95% CI=−1.5 to 57.3%). In 18 (60%) cases, an adequate pathologic diagnosis was made shortly after the first presentation. For the purpose of data analysis, an interval of more than 4 months was considered as a delay, which occurred in 12 out of 30 (40%) cases. Data were not available for one patient. The median delay between first symptom and diagnosis was 20 months (range: 0–408 months). Women with a correct diagnosis ⩽4 months after the first presenting symptom had stage I disease in 72% when compared to 75% when delay in diagnosis exceeded 4 months (differences=−2.8, 95% CI=−30.9 to 29.3). When an arbitrarily cut-off value of 12 or 24 months was used to define a delay, differences were 9.5% (95% CI=−20.6 to 43.4%) and 16.0% (95% CI=−18.3 to 55.0%; Table 1).

Surgical treatment included hysterectomy with or without BSO in 23 out of 31 (74%) and 8 out of 31 (26%) cases, respectively. Among operated women, 3 out of 31 (9%) had a thorough lymphadenectomy (resection of 19, 31 and 33 nodes), whereas 3 out of 31 (9%) had a lymph node sampling (resection of 1, 2 and 3 nodes). In one case, which was diagnosed postpartum, obvious nodal involvement was encountered. In fact, the external iliac vessels and obturator space were involved with tumour that we considered as nodal involvement. Debulking surgery was performed in 6 out of 31 (19%). Stage distribution among all 31 cases was as follows: 22 stage I, 4 stage III and 5 stage IV. Metastatic sites were mainly pelvic and transperitoneal, whereas one patient presented with lung metastasis.

Dosages from 10, 200, 500 and 1000 mg medroxyprogesterone acetate were used, whereas one patient used 10 mg norethisterone acetate. Adjuvant hormonal treatment, defined as a minimum of 200 mg medroxyprogesterone acetate, was administered in 2 out of 22 (9%) and 5 out of 9 (56%) of cases with stages I and III–IV, respectively. Two patients, both stage I, received adjuvant 10 mg medroxyprogesterone acetate or norethisterone acetate. One of these patients recurred.

The mean length of follow-up for women with and without recurrence was 62 months (median 46.5 months, range: 7–144) and 64 months (median 56, range: 2–147), respectively. Actually, 23 out of 31 (74%) patients show no evidence of disease, 5 out of 31 (16%) are dead of disease and 3 out of 31 (10%) are alive with evidence of disease.

Among stage I women undergoing hormonal treatment with or without BSO, 3 out of 15 (20%) and 1 out of 7 (14%) relapsed, respectively (differences=5.7%, 95% CI=−33.5 to 33.5%; Table 2). Limiting the analysis to stage I premenopausal women undergoing hysterectomy with or without BSO, relapse occurred in 3 out of 12 (25%) and 1 out of 6 (17%) women, respectively (differences=8.3%, 95% CI=−34.5 to 39.7%).

Women with hormonal treatment (oral contraceptives or hormonal replacement) or not at diagnosis, recurred in 0 out of 7 (0%) and 8 out of 24 (33%) (differences=33.3%, 95% CI=−53.3 to 5.3) and survived in 7 out of 7 (100%) and 19 out of 24 (79%) (differences=20.8%, 95% CI=−16.5 to 40.5) of cases, respectively (Table 2). Since all seven women with hormonal treatment at diagnosis were stage I, the positive effect of prediagnostic hormonal intake appears to be stage related (Table 1).

Among stage I women receiving adjuvant hormonal treatment or not, 0 out of 2 (0%) and 4 out of 20 (20%) relapsed, respectively (differences=−20.0%, 95% CI=−46.8 to 41.6%). Among stages III–IV women receiving adjuvant hormonal treatment or not, 1 out of 5 (20%) and 3 out of 4 (75%) relapsed, respectively (differences=−55.0, 95% CI=−81.2 to 6.8). For stages III–IV patients, survival was observed in 4 out of 5 (80%) and 2 out of 4 (50%; differences=30.0%, 95% CI=−25.0 to 68.6%), respectively.

Altogether, 8 out of 31 (26%) patients experienced one or multiple recurrences. Site of recurrence included abdominal (4 out of 31; 13%), intraperitoneal pelvic (2 out of 31; 6%), vagina (1 out of 31; 3%) or lungs (2 out of 31; 6%). A retroperitoneal recurrence occurred in 1 out of 31 (3%) of all cases and in 1 out of 8 (12%) of recurrences. However, this latter woman later also had recurrence in lungs and intra-abdominally.

**Figure 1:**
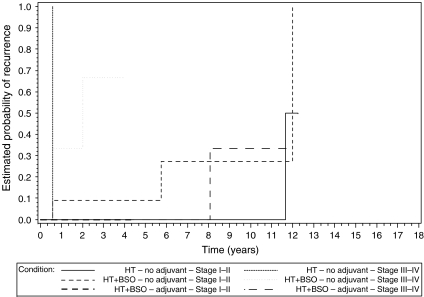
Kaplan–Meier plots show the estimated probability of recurrence in function of adjuvant hormonal treatment, type of surgery and stage.

When comparing stage I ESS without adjuvant hormonal treatment, women with or without BSO showed similar recurrence rates as shown in the Kaplan–Meier curves (Figure 1). These results suggest hormonal castration not to be beneficial for women with early-stage ESS.

**Figure 2:**
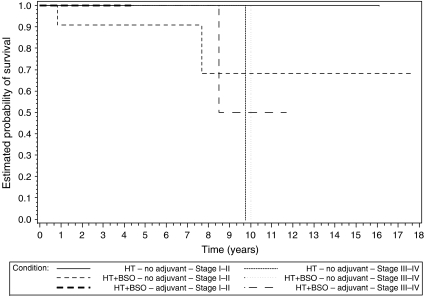
Kaplan–Meier plots show the estimated probability of survival in function of type of adjuvant hormonal treatment, type of surgery and stage.

Kaplan–Meier curves showed lower recurrence and higher survival numbers for women with stages III–IV ESS receiving adjuvant hormonal treatment than for women with stages III–IV ESS without adjuvant treatment (Figures 1 and 2). Women with stage I ESS who did not receive adjuvant hormonal treatment had a comparable recurrence rate when compared to stages III–IV women having received adjuvant hormonal treatment. Recurrence and survival data were worse among women with stage I ESS who did not take hormones at diagnosis (data not shown), suggesting that hormonal intake at diagnosis at least did not worsen the outcome.

## DISCUSSION

Although hysterectomy is the cornerstone in the treatment of ESS, the need for a radical and complete surgical staging procedure is clinically important but poorly studied. Although our results are based on retrospective findings and are in small numbers, we found no improved outcome for women with early-stage disease undergoing surgical castration and pelvic lymphadenectomy. These data suggest that the decision to perform an extended surgical procedure should be discussed with the patient and taken on an individual basis.

In the current practice, BSO is frequently added to the hysterectomy given the hormone receptor positivity and given a tendency towards recurrence even in early-stage disease. Especially in young women, menopausal symptoms after surgical castration importantly affect quality of life. Given the detrimental effect of hormone replacement in two small series (Chu *et al*, 2003; Pink *et al*, 2006), clinicians are reluctant to prescribe hormonal treatment in women with a history of ESS and symptoms of hormonal depletion. Therefore, the merits of concurrent surgical castration need to be verified.

Although the results do not differ when postmenopausal women were included (Table 2), stages I–II premenopausal women undergoing hysterectomy with or without BSO relapsed in 3 out of 12 (25%) and 1 out of 6 (17%), respectively. Numbers are too small to make final conclusions, but BSO therefore does not seem to improve the outcome. Comparing 12 premenopausal women with ESS who did not undergo BSO with 24 matched controls, Li *et al* (2005) observed similar progression-free survival and overall survival in their series. Also Gadducci *et al* (1996) and Chu *et al* (2003) observed comparable recurrence rates in women with and without BSO. Thus, since current studies cannot demonstrate a benefit for women undergoing hormonal castration for a hormone-sensitive disease and given the side effects of hormonal castration, it appears advisable to leave the ovaries *in situ* when ESS is diagnosed. Especially in young premenopausal women, this message is of clinical importance.

Kaplan–Meier plots illustrate the impact of type of surgery (hysterectomy with or without BSO) and adjuvant hormonal treatment for early- and advanced-stage disease (Figures 1 and 2). From this analysis, it appears that women with advanced-stage disease benefit from adjuvant hormonal treatment. Although this message seems obvious, this was not reported before. Only two women with early-stage ESS took adjuvant hormones. Surprisingly, women with early-stage disease not receiving adjuvant hormonal treatment had a comparable recurrence rate when compared to women with stages III–IV disease with adjuvant hormonal treatment. The observation that for recurrent disease, adjuvant hormonal treatment can compensate for stage is remarkable. These observations underscore the hormonal sensitivity of ESS. Given the considerable recurrence rates, these results suggest adjuvant hormonal treatment to be beneficial in early- and advanced-stage ESS. This hypothesis is in line with the observation that 6 out of 8 (75%) and 2 out of 7 (29%) recurrences were noted in early-stage women with and without adjuvant hormonal treatment, respectively (Chu *et al*, 2003). Given the paucity of retrospective data and the difficulties in organising prospective trials in ESS, the importance of adjuvant hormonal intake is clinically relevant.

The apparent paradox between the absence of a benefit of hormonal castration and clinical benefit of adjuvant progestins can probably be explained by a dosage effect. Daily dosages of 160 mg megestrolacetate (Chu *et al*, 2003) or 250 mg medroxyprogesteroneacetate are considered high dose, whereas physiologic effects of both drugs are already obtained with 5 mg dosages.

We challenged the role of lymphadenectomy in primary surgery. Recently, Riopel *et al* (2005), reported 5 out of 15 patients to have involved nodes somewhere in the course of their disease. Looking into detail in these data, 4 out of 5 cases with positive nodes occurred in women with primary advanced-stage (*n*=2) or recurrent disease (*n*=2). Reich *et al* (2005) reported on two cases with recurrent disease in the lymphatic system. These data suggest a higher than expected percentage of nodal involvement in advanced or recurrent ESS that might necessitate retroperitoneal exploration in this subgroup.

Data in early-stage disease are however different. Riopel *et al* (2005) mention only one case with disease otherwise limited to the uterus and positive nodes. Worthy of note, there was an interval of 3 months between hysterectomy and the second surgery during which the positive node was found. Interestingly, the case with nodal involvement at presentation from the current series was diagnosed after a postpartum haemorrhage and, in this case, nodal involvement at the obturator level was diagnosed 4 weeks after delivery (Leunen *et al*, 2003). It is intriguing to hypothesise that uterine manipulation (Riopel *et al*, 2005) or activity (Leunen *et al*, 2003) might contribute to nodal involvement in ESS otherwise limited to the uterus.

In the current study, a thorough lymphadenectomy or lymph node sampling was performed in a minority of cases only (three and three patients, respectively). Only in the case diagnosed shortly after delivery, nodal involvement was noted (Leunen *et al*, 2003). With regard to recurrence in the lymphatic system, only 1 out of 31 (3%) patient in this study developed a retroperitoneal pelvic recurrence. However, this patient subsequently developed lung- and intra-abdominal metastases. The low retroperitoneal recurrence rate (3%) in our study and the tendency of ESS to recur at different sites (Chang *et al*, 1990) suggest systematic lymphadenectomy to bear little clinical benefit in early-stage ESS.

Tumours arising in young women reduce the serendipity (oncological mindset) of the pathologist, and this adds to the diagnostic problem and delay in diagnosis (Amant *et al*, 2003). Surprisingly, in this series, however, delay in diagnosis did not alter stage distribution (stages I *vs* III–IV; Table 1). Also surprisingly, when a definition of 12 and 24 months was used to define a delay, stage differences were small (Table 2). Probably, the absence of impact of delay in diagnosis on stage distribution is explained by the indolent nature of disease (significant numbers of ESS present with early-stage disease, even when delay in diagnosis exceeds 24 months; Table 1) and the tendency for transperitoneal spread (asymptomatic spread results in considerable advanced-stage ESS at presentation; Table 1).

Also in the survival analysis, a relative good outcome is noted for stages III–IV ESS (Figure 2). Given the hormone sensitivity and the indolent nature of ESS, we consider ESS as a chronic disorder for which we apply repetitive surgery. We perform secondary and tertiary debulking surgery with organ resection and thoracotomies to tackle recurrent disease. Progestins (Gloor *et al*, 1982), gonadotropin-releasing agonists (Burke and Hickey, 2004) and aromatase inhibitors (Maluf *et al*, 2001; Leunen *et al*, 2004) are prescribed as systemic treatment in between the surgeries.

Probably, some extrapolations can be made to uterine adenosarcoma. Uterine adenosarcoma was first described by Clement and Scully in 1974 (Clement and Scully, 1974). These rare neoplasms have a benign epithelial component, whereas the stromal component typically is of low grade. In 56% of the cases, the sarcomatous component was ESS alone and in an additional 9% of cases a mixture of ESS and fibrosarcoma was present (Clement and Scully, 1990). In uterine adenosarcoma lacking sarcomatous overgrowth, we documented that in 18 out of 20 (90%) of the cases either the oestrogen or the progesterone receptor stained positive in the sarcomatous component (Amant *et al*, 2004b). Given a histological and biological similarity, we propose to extrapolate the results in ESS to uterine adenosarcoma without sarcomatous overgrowth.

## CONCLUSIONS

Endometrial stromal sarcoma is an indolent uterine tumour for which primary surgery including hysterectomy with BSO is regarded the standard primary treatment. However, in our retrospective case series, BSO did not reduce recurrence rate in premenopausal stage I women. These data suggest that ovarian preservation in young women with low-stage ESS should be discussed on an individual basis with the patient. Our data also suggest that pelvic lymphadenectomy bears little clinical benefit. Our data show high-recurrence rates in early-stage ESS, supporting the current practice in some centres to administer adjuvant hormonal treatment.

## Figures and Tables

**Table 1:** Comparison of clinical findings according to surgical stage

	**Stage I**	**Stages III–IV**
Hormonal treatment^a^ at diagnosis	7/22 (31.8)	0/9 (0)
		
*Delay of diagnosis (months)*		
⩽4	13/18 (72)	5/18 (28)
>4	9/12 (75)	3/12 (25)
⩽12	16/21 (76)	5/21 (24)
>12	6/9 (67)	3/9 (33)
⩽24	19/25 (76)	6/25 (34)
>24	3/5 (60)	2/5 (40)
Hysterectomy with BSO	15/22 (68)	8/9 (89)
Hormonal treatment after surgery	2/22 (9.1)	5/9 (56)

BSO=bilateral salpingo-oophorectomy.

Results are presented as *n* (%).

a Oral contraception or hormonal replacement.

**Table 2:** Impact of the intake of hormonal treatment, addition of bilateral salpingo-oophorectomy (BSO) and adjuvant hormonal treatment on recurrence and survival

	**Recurrence**		**Survival**	
	**Yes**	**No**	**Yes**	**No**
*Hormonal treatment at diagnosis*				
Yes	0/0 (0)	7/7 (100)	7/7 (100)	0/0 (0)
No	8/24 (37)	16/24 (63)	19/24 (79)	5/23 (21)
				
*BSO*				
No	2/8 (25)	6/8 (75)	7/8 (87)	1/8 (13)
Yes	6/23 (26)	17/23 (74)	19/23 (83)	4/23 (17)
				
*Adjuvant hormonal treatment*				
Yes	1/7 (14)	6/7 (86)	6/7 (86)	1/7 (14)
No	7/24 (29)	17/24 (71)	20/24 (83)	4/24 (17)

Results are presented as *n* (%).

## References

[bib1] Amant F, Moerman P, Cadron I, Neven P, Berteloot P, Vergote I (2003) The diagnostic problem of endometrial stromal sarcoma: report on 6 cases. Gynecol Oncol 90: 37–43 12821339 10.1016/s0090-8258(03)00207-5

[bib2] Amant F, Moerman P, Neven P, Timmerman D, Van Limbergen E, Vergote I (2005) Endometrial cancer. Lancet 366: 491–505 16084259 10.1016/S0140-6736(05)67063-8

[bib3] Amant F, Moerman P, Vergote I (2004a) The classification of a uterine sarcoma as ‘high-grade endometrial stromal sarcoma’ should be abandoned. Gynecol Oncol 95: 412–413 15491769 10.1016/j.ygyno.2004.07.021

[bib4] Amant F, Schurmans K, Steenkiste E, Verbist L, Abeler V, Tulunay G, de Jonge E, Massuger L, Moerman P, Vergote I (2004b) Immunohistochemical determination of estrogen and progesterone receptor positivity in uterine adenosarcoma. Gynecol Oncol 93: 680–685 15196864 10.1016/j.ygyno.2004.03.021

[bib5] Beer TW, Buchanan R, Buckley CH (1995) Uterine stromal sarcoma following tamoxifen treatment. J Clin Pathol 48: 596 10.1136/jcp.48.6.596-b PMC502703 7665715

[bib6] Burke C, Hickey K (2004) Treatment of endometrial stromal sarcoma with a gonadotropin-releasing hormone analogue. Obstet Gynecol 104: 1182–1184 15516445 10.1097/01.AOG.0000133533.05148.aa

[bib7] Chang K, Crabtree G, Lim-Tan S, Kempson R, Hendrickson M (1990) Primary uterine endometrial stromal neoplasms. Am J Surg Pathol 14: 415–439 2327549 10.1097/00000478-199005000-00002

[bib8] Chu M, Mor G, Lim C, Zheng W, Parkash V, Schwartz P (2003) Low-grade endometrial stromal sarcoma: hormonal aspects. Gynecol Oncol 90: 170–176 12821359 10.1016/s0090-8258(03)00258-0

[bib9] Clement P, Scully R (1974) Mullerian adenosarcoma of the uterus: a clinicopathologic analysis of ten cases of a distinctive type of Mullerian mixed tumor. Cancer 34: 1138–1149 4371193 10.1002/1097-0142(197410)34:4<1138::aid-cncr2820340425>3.0.co;2-9

[bib10] Clement P, Scully R (1990) Mullerian adenosarcoma of the uterus: a clinicopathologic analysis of 100 cases with a review of the literature. Hum Pathol 21: 363–381 2156771 10.1016/0046-8177(90)90198-e

[bib11] Evans H (1982) Endometrial stromal sarcoma and poorly differentiated endometrial sarcoma. Cancer 50: 2170–2182 7127257 10.1002/1097-0142(19821115)50:10<2170::aid-cncr2820501033>3.0.co;2-k

[bib12] Gadducci A, Sartori E, Landoni F, Zola P, Maggino T, Urgesi A, Lissoni A, Losa G, Fanucchi A (1996) Endometrial stromal sarcoma: analysis of treatment failures and survival. Gynecol Oncol 63: 247–253 8910635 10.1006/gyno.1996.0314

[bib13] Gloor E, Schnyder P, Cikes M, Hofstetter J, Cordy R, Burnier K (1982) Endolymphatic stromal myosis: surgical and hormonal treatment of extensive abdominal recurrence 20 years after hysterectomy. Cancer 50: 1888–1889 7116313 10.1002/1097-0142(19821101)50:9<1888::aid-cncr2820500940>3.0.co;2-k

[bib14] Hart W, Yoonnessi M (1977) Endometrial stromatosis of the uterus. Obstet Gynecol 49: 393–403 854242

[bib15] Kaplan E, Meier P (1958) Nonparametric estimation from incomplete observations. J Am Stat Assoc 53: 457–481

[bib16] Leunen K, Amant F, Debiec-Rychter M, Croes R, Hagemeijer A, Schoenmakers E, Vergote I (2003) Endometrial stromal sarcoma presenting as postpartum haemorrhage: report of a case with a sole t(10;17)(q22;p13) translocation. Gynecol Oncol 91: 265–271 14529693 10.1016/s0090-8258(03)00477-3

[bib17] Leunen M, Breugelmans H, De Sutter Ph, Bourgain C, Amy JJ (2004) Low-grade endometrial stromal sarcoma treated with the aromatase inhibitor Letrozole. Gynecol Oncol 95: 769–771 15582003 10.1016/j.ygyno.2004.07.063

[bib18] Li A, Giuntoli R, Drake R, Byun S, Rojas F, Barbuto D, Klipfel N, Edmonds P, Miller D, Karlan B (2005) Ovarian preservation in stage I low-grade endometrial stromal sarcomas. Obstet Gynecol 106: 1304–1308 16319256 10.1097/01.AOG.0000185511.91694.1e

[bib19] Maluf F, Sabbatini P, Schwartz L, Xia F, Aghajanian C (2001) Endometrial stromal sarcoma: objective response to letrozole. Gynecol Oncol 82: 384–388 11531300 10.1006/gyno.2001.6238

[bib20] Newcombe RG (1998) Interval estimation for the difference between independent proportions: comparison of eleven methods. Stat Med 17: 873–890 9595617 10.1002/(sici)1097-0258(19980430)17:8<873::aid-sim779>3.0.co;2-i

[bib21] Oliva E, Clement P, Young R (2000) Endometrial stromal tumors: an update on a group of tumors with a protean phenotype. Adv Anat Pathol 7: 257–281 10976906 10.1097/00125480-200007050-00001

[bib22] Pink D, Lindner T, Mrozek A, Kretzschmar A, Thuss-Patience P, Dörken B, Reichardt P (2006) Harm or benefit of hormonal treatment in metastatic low-grade endometrial stromal sarcoma: single center experience with 10 cases and review of the literature. Gynecol Oncol 101: 464–469 16368128 10.1016/j.ygyno.2005.11.010

[bib23] Reich O, Regauer S, Urdl W, Lahousen M, Winter R (2000) Expression of oestrogen and progesterone receptors in low-grade endometrial stromal sarcomas. Br J Cancer 82: 1030–1034 10737385 10.1054/bjoc.1999.1038 PMC2374426

[bib24] Reich O, Winter R, Regauer S (2005) Should lymphadenectomy be performed in patients with endometrial stromal sarcoma? Gynecol Oncol 97: 982 15944001 10.1016/j.ygyno.2005.01.034

[bib25] Riopel J, Plante M, Renaud M, Roy M, Têtu B (2005) Lymph node metastases in low-grade endometrial stromal sarcoma. Gynecol Oncol 96: 402–406 15661228 10.1016/j.ygyno.2004.10.021

[bib26] Sterne JA, Davey Smith G (2001) Sifting the evidence – what's wrong with significance tests. BMJ 322: 226–231 11159626 10.1136/bmj.322.7280.226 PMC1119478

